# Statistical Integration of ‘Omics Data Increases Biological Knowledge Extracted from Metabolomics Data: Application to Intestinal Exposure to the Mycotoxin Deoxynivalenol

**DOI:** 10.3390/metabo11060407

**Published:** 2021-06-21

**Authors:** Marie Tremblay-Franco, Cécile Canlet, Philippe Pinton, Yannick Lippi, Roselyne Gautier, Claire Naylies, Manon Neves, Isabelle P. Oswald, Laurent Debrauwer, Imourana Alassane-Kpembi

**Affiliations:** 1 Toxalim (Research Center in Food Toxicology), Toulouse University, INRAE, ENVT, INP-Purpan, UPS, 31027 Toulouse, France; cecile.canlet@inrae.fr (C.C.); philippe.pinton@inrae.fr (P.P.); yannick.lippi@inrae.fr (Y.L.); roselyne.gautier@inrae.fr (R.G.); claire.naylies@inrae.fr (C.N.); manon.neves@inrae.fr (M.N.); isabelle.oswald@inrae.fr (I.P.O.); laurent.debrauwer@inrae.fr (L.D.); 2 Metatoul-AXIOM Platform, MetaboHUB, Toxalim, INRAE, 31027 Toulouse, France; 3 Department of Veterinary Biomedicine, Faculty of Veterinary Medicine, Université de Montréal, Saint-Hyacinthe, QC J2S 2M2, Canada; imourana.alassane-kpembi@umontreal.ca

**Keywords:** mycotoxin exposure 1, transcriptomics 2, metabolomics 3, statistical integration 4

## Abstract

The effects of low doses of toxicants are often subtle and information extracted from metabolomic data alone may not always be sufficient. As end products of enzymatic reactions, metabolites represent the final phenotypic expression of an organism and can also reflect gene expression changes caused by this exposure. Therefore, the integration of metabolomic and transcriptomic data could improve the extracted biological knowledge on these toxicants induced disruptions. In the present study, we applied statistical integration tools to metabolomic and transcriptomic data obtained from jejunal explants of pigs exposed to the food contaminant, deoxynivalenol (DON). Canonical correlation analysis (CCA) and self-organizing map (SOM) were compared for the identification of correlated transcriptomic and metabolomic features, and O2-PLS was used to model the relationship between exposure and selected features. The integration of both ‘omics data increased the number of discriminant metabolites discovered (39) by about 10 times compared to the analysis of the metabolomic dataset alone (3). Besides the disturbance of energy metabolism previously reported, assessing correlations between both functional levels revealed several other types of damage linked to the intestinal exposure to DON, including the alteration of protein synthesis, oxidative stress, and inflammasome activation. This confirms the added value of integration to enrich the biological knowledge extracted from metabolomics.

## 1. Introduction

Mycotoxins are toxic fungal secondary metabolites frequently found as contaminants of food and feed. Among them, deoxynivalenol (DON) is very a prevalent mycotoxin in cereals and cereal products [1,2]. It is one of the most frequently occurring contaminants in human and animal diets. The intestine constitutes the first biological barrier to ingested toxics, and is therefore the first target of DON [3]. Several studies have demonstrated the adverse effects of DON on the intestine, such as impaired immune function, the inhibition of intestinal nutrient absorption, and altered intestinal cell and barrier functions [3,4,5]. Due to their cereal-rich diet, pigs are particularly exposed to DON. In addition, they can be considered good models for extrapolating to humans, with a digestive physiology very similar to that of humans [3]. Pigs therefore constitute a well-suited model to assess the effects of DON on intestinal health.

Since the metabolome responds to stress long before standard biomarkers do, metabolic fingerprinting was previously used to address the subacute exposure to DON at a concentration that is likely to occur in food or feed [6,7]. The effects of low doses of DON, like those of many other toxicants, are often subtle, and confounding variability (due to experimentation, analytics, or physiology) can hamper variations caused by the toxicological exposure. It is therefore difficult to adjust models discriminating control from exposed individuals using the metabolomic data matrix alone [8]. Consequently, only a few metabolites were found to be discriminant in the above-mentioned study, and the extracted biological knowledge was quite limited.

Taking advantage of recent advances in high-throughput analytical techniques, many studies integrate the use of different ‘omics platforms on the same samples, which makes it possible to have a set of data at the genome scale, while being able to measure biomolecules at the different functional levels of the studied organisms [9,10]. This ‘omics data fusion allows us to handle a biological system as a whole to gain insight into its complex functioning through the identification of more informative models [11]. The main statistical methods used to integrate several ‘omics datasets are the penalized canonical correlation analysis (CCA) [12,13] and self-organizing map (SOM) [14,15], used to compute correlations between blocks, and the multi-block generalizations of partial least squares (PLS) to fit a model between a biological factor and ‘omics profiles [16,17,18,19,20,21,22,23].

This study aimed to assess whether the NMR-based metabolomic modeling of DON exposure can be enriched by integrating complementary information contained in transcriptomic data, based on a combination of sparse CCA and SOM analysis, to select within- and between-blocks-correlated features, and the O2-PLS to model the selected features. Furthermore, we aimed at assessing the correlations between these two functional levels to identify genes and metabolites as markers of exposure to the mycotoxin. To this end, we generated transcriptomic and metabolomic datasets from pig intestinal explants exposed to a similar and low dose of DON.

## 2. Results

In the present study, metabolomic and transcriptomic data were generated from 16 jejunal explants treated (n = 8) or not (n = 8) with 10 µM DON for 4 h to assess the effect of this toxin at the intestinal level.

### 2.1. Individual PLS-DA Modeling of ‘Omics Data

#### 2.1.1. Metabolomic Effect of DON on Intestinal Explants

We first analyzed effect of DON on the metabolome. Noisy NMR features were removed prior to analysis because the noise-related variability hampered us from seeing the biological variability and prevented us from fitting the models (NMR data used in this study are presented in file S1).

**Figure 1:**
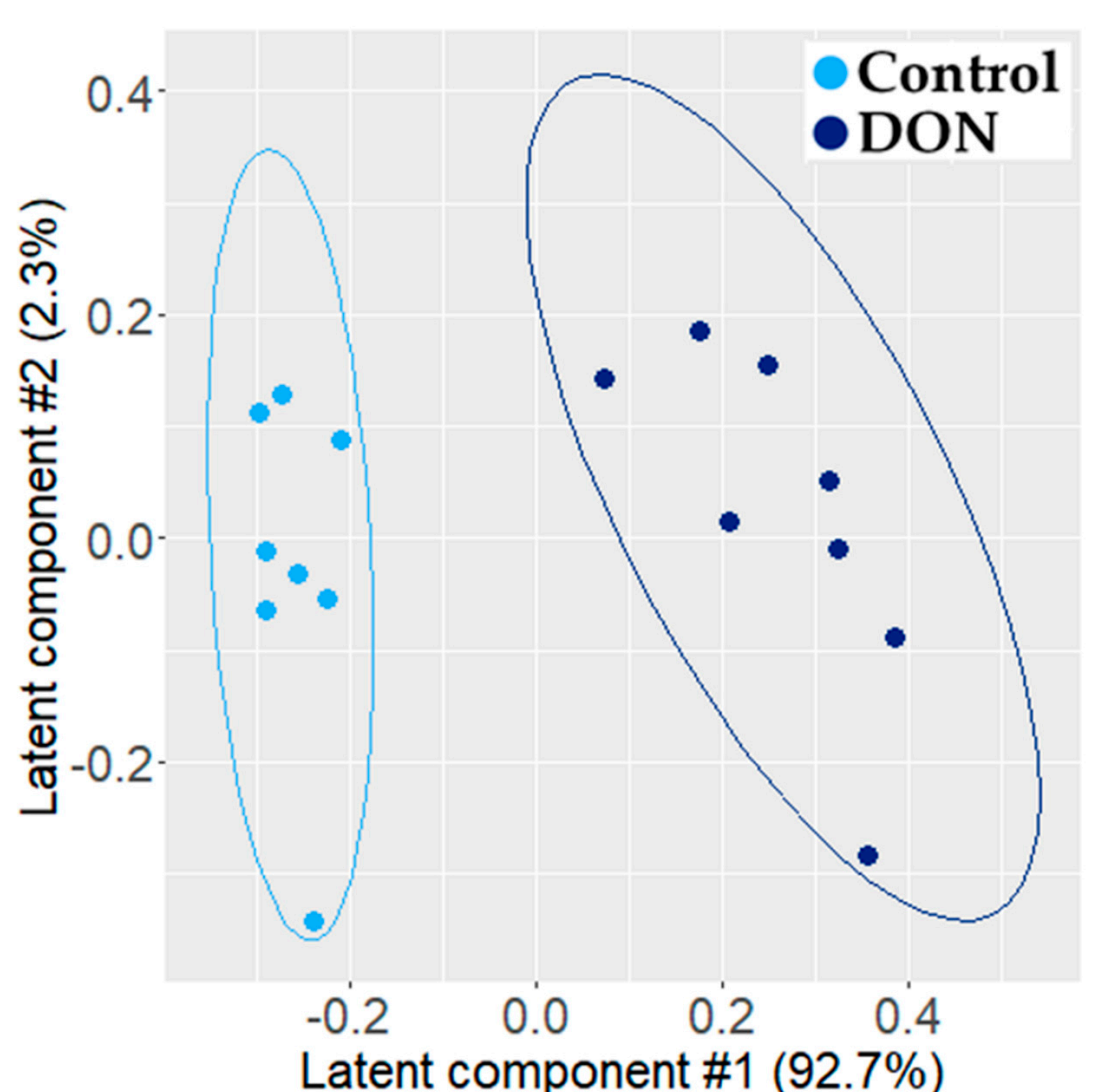
Two-dimensional PLS-DA (R2 = 95% and Q2 = 0.91) score plot of the integrated 1H NMR spectra of jejunum explant extracts (control, n = 8, light blue dot; DON-exposed, n = 8, dark blue dot). Each dot corresponds to an individual.

**Figure 2:**
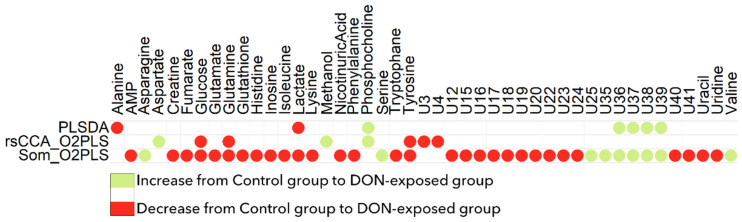
Comparison of metabolites found to be discriminant by PLS-DA (upper line), Robust sparse CCA+O2-PLS-DA (middle line), or SOM+O2-PLS-DA (lower line).

A valid and robust PLS-DA model (A = 2 latent components, percentage of explained variance: R2 = 95%; predictive capacity: Q2 = 0.91; permutation test: *p* = 0.005) was fitted on the Pareto-scaled metabolomic data. Figure 1 presents the projection of individuals onto the first latent plane. This score plot showed a clear separation between control and exposed individuals. Sixteen NMR features had a VIP (variable importance in the projection) value > 1.0 and were statistically different at the 5% threshold (false discovery rate-corrected *p*-value from the Wilcoxon test). These corresponded to three metabolites: alanine, lactic acid, and phosphocholine (see Figure 2).

#### 2.1.2. Transcriptomic Effect of DON on Intestinal Explant

We next analyzed the transcriptomic response of the intestinal explants treated or not with 10 µM of DON for 4 h. Due to computational limitations, the number of transcriptomic features had to be limited and was set to 15,000, based on the highest standard deviation.

**Figure 3:**
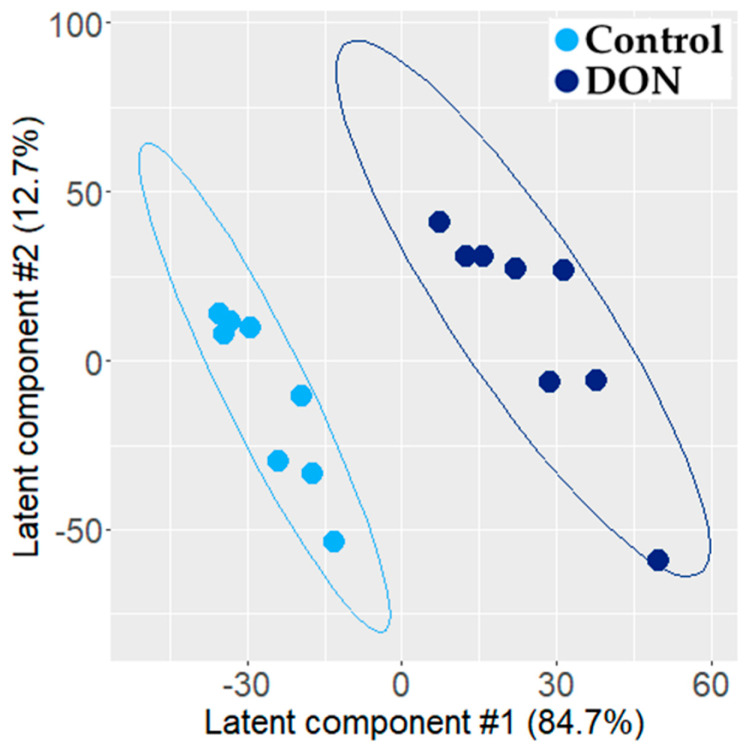
Two-dimensional PLS-DA (R2 = 95% and Q2 = 0.91) score plot of transcriptomic data generated from jejunum explant extracts (control, n = 8, light blue dot; DON-exposed, n = 8, dark blue dot). Each dot corresponds to an individual.

The analysis of Pareto-scaled transcriptomic data also generated a valid and robust PLS-DA model (A = 2 latent components, R2 = 97.3% and Q2 = 0.81, permutation test: *p* = 0.005). The score plot of the PLS-DA model showed a clear separation between the control and exposed individuals (Figure 3). A total of 1468 probes had a VIP value > 1.0 and were significantly different at the 5% threshold (false discovery rate-corrected *p*-value from the Wilcoxon test), corresponding to 1094 upregulated and 374 downregulated genes. The cytokine genes *CSF2*, *TNF*, *IL-17A*, and *IL-22*, the genes encoding the interleukins 1 alpha and beta (*IL-1α* and *IL-1β*), chemokine ligand 20 (*CCL20*), and the Nuclear Factor Kappa B Subunit 1 (*NF-κB1*) were the most upregulated genes, as well as the prostaglandin-endoperoxide synthase (*PTGS*).

### 2.2. Fusion of Transcriptomic and Metabolomic Data

In order to extract more information from metabolomics, a statistical fusion of both ‘omics datasets was performed. Robust sparse CCA (rsCCA) and dissimilarity-based SOM were first applied to select correlated features and O2-PLS was then performed using each set of selected features to model the relationship between DON exposure and ‘omics profiles.

#### 2.2.1. Combination of Robust Sparse CCA and O2-PLS

**Figure 4:**
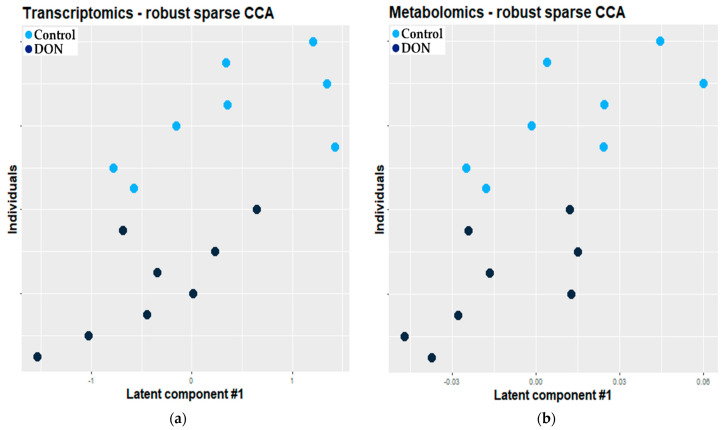
Projection of individuals along the first latent variable of the O2-PLS-DA model based on the features selected by rsCCA (light blue dot: control, n = 8; dark blue dot: exposed, n = 8). Each dot corresponds to an individual. (**a**) Transcriptomic dataset; (**b**) metabolomic dataset.

Twelve transcriptomic and 12 metabolomic features were selected by rsCCA. An O2-PLS model was then fitted using this set of features. The constructed model explained 60.4% of the total variability and showed a very low error of prediction (0.01). Figure 4 presents the projection of individuals along the first latent variable of the fitted O2-PLS-DA model for the transcriptomic (a) and metabolomic (b) datasets, respectively. Both figures showed that the observations were not well discriminated according to the exposure to DON.

All the correlated transcriptomic (12) and metabolomic (12) features were found to be discriminant in the O2-PLS model. Metabolomic features corresponded to six identified metabolites (see Figure 2); the aspartate, methanol, and phosphocholine concentrations were lower in the control group, whereas glucose, glutamine, and tyrosine concentrations were higher in the control group.

#### 2.2.2. Combination of Self Organizing Map and O2-PLS

**Figure 5:**
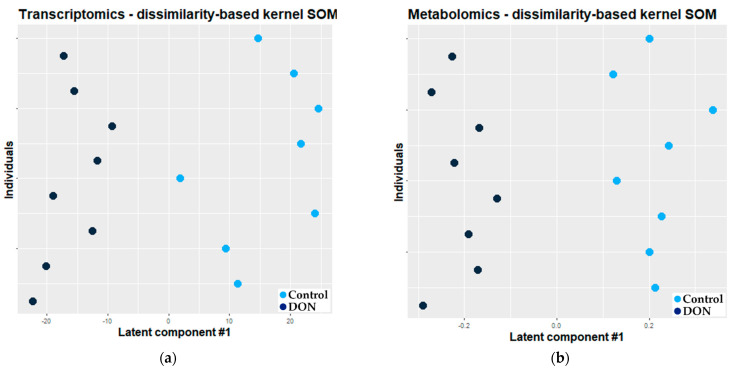
Projection of individuals along the first latent variable of the O2-PLS-DA model based on the features selected by dissimilarity kernel-based SOM (light blue dot: control, n = 8; dark blue dot: exposed, n = 8). Each dot corresponds to an individual. (**a**) Transcriptomic dataset; (**b**) metabolomic dataset.

A total of 11,069 transcriptomics and 154 metabolomics features were selected by the dissimilarity kernel-based SOM. The adjusted O2-PLS model based on these selected features explained half of the total variability and showed a very low error of prediction (0.01). Figure 5 presents the projection of individuals along the first latent variable of the fitted O2-PLS model for the transcriptomic (a) and metabolomic (b) datasets, respectively. In the case of the model based on features selected by dissimilarity kernel-based SOM, the observations were well discriminated according to the exposure.

The analysis revealed that 1443 transcriptomic features and 39 metabolites (among which 21 have been identified) were discriminant (see Figure 2, last line for metabolites). The concentrations of AMP, creatine, fumarate, glucose, glutamate, glutamine, glutathione, histidine, inosine, isoleucine, lactate, lysine, nicotinuric acid, phenylalanine, tryptophan, tyrosine, uracil, and uridine were higher in the control group, whereas the concentrations of asparagine, serine, and valine were lower in the control group. The use of the MetExplore metabolic network analysis tool (right-tailed Fisher test with Bonferroni’s multiple test correction) showed that the metabolic pathways significantly enriched in this set of discriminant metabolites are primarily linked to the aminoacyl-tRNA biosynthesis and the metabolism of amino acids and 2-oxocarboxylic acids (Table 1).

**Figure 6:**
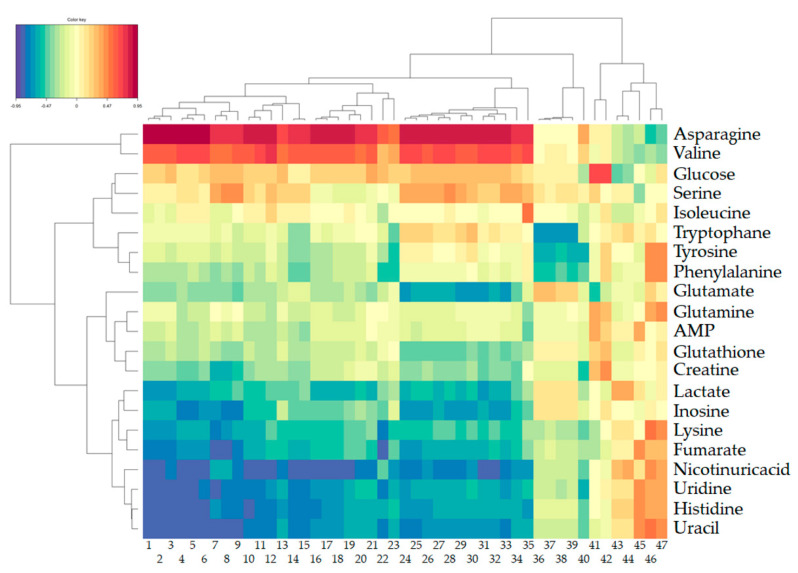
Correlations between the discriminant metabolites and transcriptomic features. Meaning of transcriptomics features (columns): 1: *IL-1α*.1; 2: *IL-1α*.3; 3: *IL-1α*.2; 4: *IL-1β*.2; 5: *IL-1β*; 6: *IL-1β*.1; 7: *IL10*.2; 8: *IL10*.1; 9: *IL10*.3; 10: *SOCS3*.1; 11: *SOCS3*.2; 12: *IL4R*.1; 13: *SOCS3*.3; 14: *CXCL2*.1; 15: *CXCL2*.2; 16: *IL8*.3; 17: *IL8*.2; 18: *IL8*.1; 19: *IL8*.4; 20: *IL22*; 21: *IL17A*; 22: *IL23A*; 23: *CXCL2*.3; 24: *TXNIP*.3; 25: *TXNIP*.2; 26: *TXNIP*.1; 27: *TXNIP*.10; 28: *TXNIP*.9; 29: *TXNIP*.8; 30: *TXNIP*.6; 31: *TXNIP*.4; 32: *TXNIP*.5; 33: *TXNIP*.7; 34: *TXNIP*.11; 35: *IL4R*.2; 36: *IL6*.3; 37: *IL6*.2; 38: *IL6*.4; 39: *IL6*.1; 40: *CYP1A1*; 41: *SOD2*; 42: CX060127; 43: *CYP3A29*.2; 44: *CYP3A29*.1; 45: *GCG*; 46: *CYBRD1*.1; 47: *CYBRD1*.2.

Figure 6 presents a heatmap of the correlations between the metabolomic and transcriptomic features, as selected by the SOM-based method and filtered by O2-PLS-DA. One particular interesting highlight of this figure includes the fact that the level of the discriminant metabolite glutathione correlates positively with the gene expression of *SOD2* and negatively with the gene expression of *TXNIP*.

## 3. Discussion

Several studies have demonstrated the adverse effects of DON, which can be observed at different ‘omics levels [24,25,26]. To the best of our knowledge, no study has combined two or more ‘omics datasets to give a more global view of disruptions due to DON exposure.

### 3.1. Individual PLS-DA Modeling of ‘Omics Data

Firstly, each ‘omics dataset was individually analyzed using PLS-DA. PLS-DA is well adapted for ‘omics data (high dimension and collinearities) because linear combinations of spectral features are constructed and few of these are used in the model.

Three metabolites (alanine, lactic acid, and phosphocholine) participated in the discrimination of DON-exposed explants from control explants. This low number of metabolites indicated that, despite a clear separation of observations, little information can be extracted from the metabolomic data. This could result from variables containing con-founding variability (experimental or instrumental variability for example), which masks biological variability [8].

To date, very few discriminant metabolites including those detected here have been reported in NMR-based metabolomics investigations of the alterations induced by DON in pigs [6,27,28]. Phosphocholine is a downstream metabolite in the catabolism of phosphatidylcholine. Cellular lactate predominantly stems from alanine and glucose through their conversion into pyruvate, and lactate homeostasis is primarily related to glucose metabolism [29]. Likewise, a crosstalk between glycolysis and the catabolism of phosphocholine has been shown for energy supply to the cell under specific situations [30]. These findings suggest that the DON exposure shifts the main energy source in the intestinal tissue from amino acids in physiological conditions [31] to glucose, and confirm the previously reported disturbance of energy metabolism as a prominent metabolic effect of the mycotoxin in pigs [28].

When looking at discriminant features of transcriptomic data, the top upregulated genes in the intestinal explants were mainly related to inflammation and immunity (cytokine genes *CSF2*, *TNF*, *IL-17A*, and *IL-22*; genes encoding the interleukins 1 alpha and beta chemokine ligand 20; and the Nuclear Factor Kappa B Subunit 1 were the most upregulated genes). Other inflammatory genes in the top upregulated category included the prostaglandin-endoperoxide synthase, also known as cyclooxygenase; the *NR3C1* gene encoding a nuclear glucocorticoid receptor involved in inflammatory responses and cellular proliferation; and *CXCL2* and *CXCR4*, which encode the C-X-C motif chemokine Ligand 2 and Receptor 4 expressed at sites of inflammation and involved in immunoregulatory and inflammatory processes. These observations are in line with previous publications pointing at inflammation as the main endpoint for the molecular toxicity of type B trichothecenes in the intestine [24,26,32,33].

### 3.2. Fusion of Transcriptomic and Metabolomic Data

Methodological issues encountered with the analysis of one single ‘omics dataset are worsened by ‘omics fusion. Some of these problems are related to the high dimensionality of matrices and biological noise. This latter can hinder the effects of the biological factor and may prevent evidencing the effects of the factor of interest. It is therefore essential to consider these problems when choosing the statistical method to apply for ‘omics integration. CCA is designed to select correlated features. However, CCA fails due to ill-, or even un-, conditioned matrices in high dimensions. A regularized method may be applied to solve this issue. Another issue is with selecting relevant and meaningful correlated features from the many thousands of features present in the original datasets, among which many may be noisy. In this study, we firstly selected correlated features using robust sparse CCA or SOM methods prior to multi-block modeling. This selection step enabled us to decrease the number of features included in the O2-PLS-DA model. By clustering objects into a bidimensional grid, the SOM algorithm takes into account possible nonlinear relations between objects, which is a great advantage over other clustering algorithms. The SOM algorithm also enables us to preserve the topology of the original data, such as the correlation structure of features in this study [34]. Moreover, the orthogonal step of O2-PLS-DA allowed us to remove variation in noisy features, if selected by sparse CCA or SOM methods.

#### 3.2.1. Combination of Robust Sparse CCA and O2-PLS

Twelve transcriptomic and 12 metabolomic features were selected by rsCCA. This low number of selected features agreed with the simulation results (simulation study is detailed in file S2). rsCCA demonstrated very high values of specificity (Figure S1), meaning that only correlated features were selected by this method. This low number of selected features could explain the fact that DON-exposed explants were not well discriminated from control explants in the O2-PLS-DA modeling in the sense that not only correlated features were markers of exposure in this study. Although the number of identified metabolites was greater than in the individual analysis of metabolomic block, this number was still very low and not sufficient to gain insight into dysregulations due to mycotoxin exposure at the organism level.

#### 3.2.2. Combination of Kernel Dissimilarity-Based SOM and O2-PLS

The heterogeneity of measurements is another issue encountered when integrating ‘omics data. To deal with this issue, we applied the SOM method to two kernel matrices—namely, dissimilarity and Gaussian kernels. A kernel matrix provides pairwise information between objects. Kernels are a widely used and flexible method to deal with com-plex data of various types [35]

We only applied a dissimilarity kernel-based SOM to the biological data because this method showed higher values for sensitivity and specificity than Gaussian kernel-based or raw data-based SOM (Figures S1 and S2).

A total of 11,069 transcriptomic and 154 metabolomic features were selected by the dissimilarity kernel-based SOM. The kernel based-SOM method selected many more features than rsCCA. This result was expected from the results of the simulation study (see Figures S2 and S3): SOM methods showed a high number of selected features, very high values of sensitivity, and very low values of specificity, meaning that these methods were able to identify correlated features but did not select only the correlated features. By not selecting only the correlated features, the SOM method enabled us to catch some features that seemed to be markers of biological effect. This could explain the best discrimination obtained using the SOM-based model. On the other hand, the “orthogonal step” of the O2-PLS modeling enables us to remove noisy variability, reducing the influence of correlated features with a low biological relevance in the modeling step.

Metabolites found to be discriminant in the O2-PLS-DA modeling corroborate previous in vivo findings in the plasma and liver of piglets exposed to DON in our lab [6]. The synthesis of aminoacyl-tRNA and the metabolism of amino acids and 2-oxocarboxylic acids contribute to the global process of protein synthesis, which inhibition is a well-characterized molecular effect of trichothecene mycotoxins including DON on eukaryotic cells [36].

The fusion of transcriptomic and metabolomic data showed that the discriminant metabolite glutathione correlates positively with the gene expression of *SOD2* and negatively with the gene expression of *TXNIP*. Gluthatione is an endogenous tripeptide that shields cellular macromolecules from oxidative stress by directly scavenging endogenous and exogenous oxidants or by recycling the antioxidant vitamins C and E [37]. The protein encoded by *SOD2* is one of two isozymes responsible for destroying free superoxide radicals in the body. *TXNIP* encodes the thioredoxin-binding protein that inhibits the antioxidative function of thioredoxin. Oxidative stress holds an important place in the mechanisms of the toxicity described for DON in eukaryotic cells, with two reported effects for this mycotoxin being the generation of reactive oxygen species and the alteration of antioxidant status [33]. *TXNIP* also participates in the activation of the *NLRP3* inflammasome, bridging oxidative stress to inflammation [38]. The connection between oxidative stress and inflammation is delineated in Figure 6 by the strong correlation between the expression of genes encoding pro-inflammatory and Th17 cytokines (*IL-1α*, *IL-1β*, *IL17A*, *IL22*, *IL23A*) and a chemokine (IL8), and the levels of amino acid metabolites asparagine and valine on the one hand and these metabolites and *TXNIP* expression on the other hand. The sensing of some amino acids including asparagine and valine has been shown to control intestinal inflammation via the integrated stress response mechanism [39,40]. We and others previously reported that DON triggers the transcription of IL1-b in the intestinal tissue [24,41]. This cytokine is produced in a proprotein form that needs to be processed to its active form by caspase 1. The activation of the *NRLFP3* inflammasome by DON and the subsequent processing and secretion of cytokines belonging to the IL-1 family was later demonstrated [32]. The strong positive correlation between the expression of *TXNIP* and the level of asparagine—and valine, to a lesser extent—is consistent with the involvement of oxidative stress in the DON induced-activation of the *NRLFP3* inflammasome.

## 4. Materials and Methods

### 4.1. Experimentation

#### 4.1.1. Chemicals

William’s Medium E, glucose and DON, were purchased from Sigma (Saint Quentin Fallavier, France). Penicillin/streptomycin, gentamycin, FBS, and amino acids (Ala/Glu) came from Eurobio (Courtaboeuf, France).

#### 4.1.2. Animals

All the experimental procedures were conducted under the approval of the French Ministry of Higher Education and Research (decision n° #6303_2016080314392462) and the Ethics committee of Pharmacology-Toxicology, Toulouse-Midi-Pyrénées in animal experimentation Toxcométhique (decision n° TOXCOM/0163/PP, 2 February 2017). Three authors (I.A.K., I.P.O., and P.P.) have an official agreement with the French Veterinary Services authorizing animal experimentation.

#### 4.1.3. Treatment of Jejunum Explants

Eight 5-week-old piglets were used to generate jejunal explants. The piglets were weaned at 4 weeks and fed *ad libitum* prior to the experiment. Explants were prepared as already described [33]. Briefly, 5 cm middle jejunum segments were collected and flushed with William’s Medium containing 4.5 g/L of glucose, 1% of penicillin/streptomycin, 0.5% of gentamycin, 10% FBS, and 30 mM of amino acids (Ala/Glu). Each segment was opened longitudinally, and multiple pieces of 6 mm diameter were obtained using biopsy punches (Centravet, Lapalisse, France).

DON was dissolved in dimethylsulfoxide (DMSO) at 60 mM, before dilution in complete culture media. Control samples were treated with equivalent concentrations of DMSO previously tested to be nontoxic for intestinal explants (data not shown). Each of two explant samples from each of eight piglets were deposited villi upward on biopsy sponges per well in 6-well plates (Cellstar, Greiner Bio-One, Frickenhausen, Germany) containing control or DON-contaminated (10 µM) medium. The explants were cultured at 39 °C under a CO_2_-controlled atmosphere with orbital shaking for 4 h. After treatment, the explants were frozen in liquid nitrogen and stored at −80 °C before transcriptional and metabolomics analysis. N = 8 control samples and n = 8 DON-contaminated were used for both transcriptomic and metabolomic analysis.

### 4.2. ‘Omics Analysis

#### 4.2.1. Transcriptomics

For gene expression analysis, total RNA was extracted using lysing matrix D tubes (MP Biomedicals, Illkirch, France) containing 1 mL of Extract-All reagent (Eurobio), following the manufacturer’s instructions. The RNA concentration and purity were determined using a NanoDrop spectrophotometer (Labtech International, Paris, France). RNA quality was assessed using an Agilent Bioanalyzer (Agilent, Les Ulis, France), and the mean (±SD) RNA integrity number (RIN) was 7.23 ± 0.55.

Gene expression profiles were analyzed at the GeT-TRiX facility (GénoToul, Génopole Toulouse Midi-Pyrénées) using Agilent Sureprint G3 Porcinet 60K_DEC2011 microarrays (8 × 60 K, design 037880) following the manufacturer’s instructions. For each sample, Cyanine-3 (Cy3)-labeled cRNA was prepared as described in Alassane-Kpembi et al. [33]. Microarray data and experimental details are available in NCBI’s Gene Expression Omnibus [42] and are accessible through GEO Series accession number GSE165968. All the data analyses were performed using R (R Core Team, 2018 [43]) and the Bioconductor package [44], as described in GEO accession GSE165968. Raw data (median signal intensity) were filtered, log2-transformed, and normalized using the quantile method [45]. The transcriptomic matrix includes n = 16 observations and *p* = 41,436 features.

#### 4.2.2. ^1^H-NMR-Based Metabolomics

Tissue samples (100 mg of tissue) were extracted in methanol/dichloromethane/water (2:2:1.4, *v*/*v*/*v*) as described by Beckonert et al. [46].

All the 1H NMR spectra were generated using a Bruker Avance III HD spectrometer (Bruker Biospin, Rheinstetten, Germany) operating at 600.13 MHz for proton resonance frequency using an inverse detection 5 mm 1H-13C-15N-31P cryoprobe. ^1^H NMR spectra of aqueous explant extracts were acquired and processed as previously described [47] using 512 transients, a spectral width of 20 ppm, and a relaxation delay of 2 s.

NMR spectra were phase- and baseline-corrected and then calibrated (TSP, 0.0 ppm) using the Topspin software (version 3.5, Bruker). Then, the NMR data were reduced using the AMIX software (version 3.9, Bruker) to integrate 0.01 ppm-wide regions corresponding to the δ 10.0–0.5 ppm. The 5.1–4.5 ppm region, which includes the water resonance, was excluded in the NMR spectra of aqueous extracts. A total of q = 753 NMR buckets were included in the data matrix. Normalization to the total spectral area was applied to each integration region to account for differences in the sample amount. The metabolomic matrix includes n = 16 observations and *p* = 753 features. To confirm the chemical structures of the metabolites of interest, 2D ^1^H-^1^H COSY (correlation spectroscopy) and 2D 1H-13C HSQC (heteronuclear single quantum coherence spectroscopy) NMR spectra were also generated for selected samples. Spectra assignment was based on matching the 1D and 2D data to reference spectra in a homemade reference database, as well as with another database (https://www.hmdb.ca/, release 3.0, accessed on 11 May 2021) and reports in the literature.

Genome-scale metabolic network (GSMN) analysis was performed on the dataset of discriminant metabolites, as previously described [48]. To this end, the Sus scrofa GSMN was imported in MetExplore web server from KEGG database (network 4105, downloaded from KEGG database 24 August 2020) [49,50]. The network contains 1807 meta-bolic reactions and 1520 metabolites.

### 4.3. Statistical Analysis

All the statistical analyses were conducted under R [43] with in-house scripts developed for this study.

#### 4.3.1. Sparse CCA Analyses

Canonical correlation analysis [51] is a method that studies the correlation relationships between two sets (or blocks) of features measured on the same individuals. Briefly, CCA maximizes the correlation between two linear combinations from the two blocks of features. The coefficients estimated for the definition of the linear combinations are used to explore the relationships between features. In this study, we used Robust Sparse CCA [13] for its ability to select relevant correlated features also in presence of outlier observations. Features with non-null loading values in the first pair of linear combinations (transcriptomic and metabolomic datasets) were selected. We adapted scripts from Wilms and Croux [13] to perform Robust Sparse CCA.

#### 4.3.2. SOM Analyses

A self-organizing map [52] is an artificial neural network learned using an unsupervised algorithm aiming at projecting and classifying objects (individuals or features) into a lower dimensional space (e.g., two-dimensional). Prototype vectors are associated with each unit of the map: these are weight vectors, used to classify objects in map units. In this study, prototypes were randomly initialized. Two steps were iteratively performed in the stochastic version of SOM algorithm. In the first step, a randomly drawn single feature was assigned to the unit whose prototype was closest to it (according to the chosen distance). The second step was an update step: prototypes of the closest unit and its neighbors were moved towards the drawn object.

In this study, SOM was used to classify features. Features clustered in the same unit were considered to be correlated. A raw matrix or kernel matrix, based on a dissimilarity matrix or Gaussian kernel, were used to learn SOM. Examples of commonly used distance metrics include Euclidean distance and correlation for relative abundance data [35].

The mixKernel [35] (https://CRAN.R-project.org/package=mixKernel, v0.3, accessed on 11 May 2021) package was used to compute the kernel matrix from the original data. The SOMbrero [53] (https://CRAN.R-project.org/package=SOMbrero, v1.2-4, accessed on 11 May 2021) package was used to perform SOM analyses on raw matrixes or kernel matrixes.

#### 4.3.3. PLS-DA Analyses

Partial least squares [54] (PLS) regression seeks a mathematical relationship between a quantitative biological factor of interest—drug doses, for example—and several features, such as spectral data. PLS-DA is an extension of PLS regression for categorical factors, DON exposure in this study.

PLS-DA analyses were conducted using the ropls package [55] (v1.16.0).

O2-PLS [54] is a generalization of the OPLS [56] regression method and can be used for combining two ‘omics datasets. It models both predictive and systematic variation. The variation present in each of the two matrices is split up into three parts. The first part corresponds to the joint—i.e., predictive (useful for the prediction of the studied biological factor)—variation. For example, in the transcriptomic data matrix, the joint variation describes expression profiles that are useful for predicting metabolite profiles. The second part is the orthogonal part (confounding variability—e.g., physiological, experimental, or technical variations). It corresponds to the unique systematic variation in the transcriptomic data matrix, for example. This part is not useful in predicting the metabolomic data. The final part corresponds to unexplained variance (noise).

The OmicsPLS package [57] (https://CRAN.R-project.org/package=OmicsPLS, v1.1.0, accessed on 11 May 2021) was used to build O2-PLS models.

## 5. Conclusions

This study had two objectives. The statistical methods sparse CCA and SOM were compared regarding their ability to select correlated features and, thus, to be used as in noise pre-filtering before O2-PLS modeling. Biologically, the information extracted from the metabolomic data could be prevented from technical variability and noise. We aimed at combining metabolomic data with transcriptomic data to enhance the information extracted from metabolomics and gain insight into effects of DON exposure on the intestine.

To apply sparse CCA, sparsity parameters have to be tuned. Moreover, due to computational limitations, not all the transcriptomic features could be included, and thus features have to be selected before applying CCA. We chose to include features displaying the highest standard deviation, even if this choice could discard discriminant features. Even with a limited number of features, this method is very time-consuming. Although some parameters (map dimensions, distance measures) also have to be set before applying SOM, this method is less time-consuming.

In this study, we showed that the integration of metabolomic data with transcriptomic data enables us to extract more information from metabolomics by combining feature selection based on SOM and an orthogonal step in PLS-DA modeling.

Indeed, we increased the number of discriminant metabolites from seven, as detected by the individual metabolomic block analysis, to 39, as selected by the dissimilarity kernel-based SOM. Both steps enabled us to remove noisy features. At the end of the process, it was possible to gain a better insight into metabolic pathways disrupted by exposure to DON. On top of the clues of the disturbance of energy metabolism previously reported in metabolomics studies, the integration of the metabolomics data and transcriptomics features simultaneously generated on jejunal explants remarkably brought out several other types of damage caused by the intestinal exposure to DON, including the alteration of protein synthesis, oxidative stress, and inflammasome trigger. In future work, we think that applying this integration to a panel of biological samples (e.g., plasma, lung, brain) could help us extend the extracted information to the whole-organism scale.

## Figures and Tables

**Table 1:** Metabolic pathways significantly enriched in DON-exposed intestinal explants.

Pathway Name	Number of Mapped Metabolites	Coverage (%)	BH-Corrected *p*-Value
Aminoacyl-tRNA biosynthesis	10	21.3	1.73 × 10^−9^
Biosynthesis of amino acids	8	12.7	9.48 × 10^−6^
Alanine, aspartate and glutamate metabolism	5	15.2	5.79 × 10^−4^
Arginine biosynthesis	3	21.4	5.18 × 10^−3^
Phenylalanine, tyrosine and tryptophan biosynthesis	2	50.0	6.48 × 10^−3^
D-Glutamine and D-glutamate metabolism	2	40.0	9.18 × 10^−3^
Nitrogen metabolism	2	33.3	0.01
2-Oxocarboxylic acid metabolism	3	14.3	0.01
Valine, leucine and isoleucine biosynthesis	2	25.0	0.018

## Data Availability

Microarray data and experimental details are available in NCBI’s Gene Expression Omnibus [42] and are accessible through GEO Series accession number GSE165968. Preprocessed NMR data are in the supplementary excel file S2.

## Supplementary Materials

The following are available online at https://www.mdpi.com/article/10.3390/metabo11060407/s1, Supplementary file S1: Excel file including preprocessed NMR data. Supplementary file S2: Details of simulation study run to compare rsCCA and SOM, combined with O2-PLS, on artificial data. Figure S1: Average specificity of SOM (dissimilarity kernel-based and raw) and Robust Sparse CCA methods calculated for transcriptomic (middle blue bars) and metabolomic (light blue bars) blocks. In total, 100 Monte Carlo simulations. Figure S2: Average sensitivity of SOM (dissimilarity kernel-based and raw) and Robust Sparse CCA methods calculated for transcriptomic (middle blue bars) and metabolomic (light blue bars) blocks. In total, 100 Monte Carlo simulations. Figure S3: Average percentage of selected features by SOM (dissimilarity kernel-based and raw) and Robust Sparse CCA methods for transcriptomic (middle blue bars) and metabolomic (light blue bars) blocks. In total, 100 Monte Carlo simulations.

## References

[1] Gruber-Dorninger C., Jenkins T., Schatzmayr G. (2019). Global Mycotoxin Occurrence in Feed: A Ten-Year Survey. Toxins.

[2] Knutsen H.K., Alexander J., Barregård L., Bignami M., Brüschweiler B., Ceccatelli S., Cottrill B., Dinovi M., Grasl-Kraupp B., Hogstrand C. (2017). Risks to Human and Animal Health Related to the Presence of Deoxynivalenol and Its Acetylated and Modified Forms in Food and Feed. EFSA J..

[3] Pinton P., Oswald I.P. (2014). Effect of Deoxynivalenol and Other Type B Trichothecenes on the Intestine: A Review. Toxins.

[4] Waché Y.J., Valat C., Postollec G., Bougeard S., Burel C., Oswald I.P., Fravalo P. (2009). Impact of Deoxynivalenol on the Intestinal Microflora of Pigs. Int. J. Mol. Sci..

[5] Pierron A., Mimoun S., Murate L.S., Loiseau N., Lippi Y., Bracarense A.-P.F.L., Schatzmayr G., He J.W., Zhou T., Moll W.-D. (2016). Microbial Biotransformation of DON: Molecular Basis for Reduced Toxicity. Sci. Rep..

[6] Alassane-Kpembi I., Canlet C., Tremblay-Franco M., Jourdan F., Chalzaviel M., Pinton P., Cossalter A.M., Achard C., Castex M., Combes S. (2020). 1H-NMR Metabolomics Response to a Realistic Diet Contamination with the Mycotoxin Deoxynivalenol: Effect of Probiotics Supplementation. Food Chem. Toxicol..

[7] O’Gorman A., Brennan L. (2017). The Role of Metabolomics in Determination of New Dietary Biomarkers. Proc. Nutr. Soc..

[8] Boccard J., Rudaz S. (2016). Exploring Omics Data from Designed Experiments Using Analysis of Variance Multiblock Orthogonal Partial Least Squares. Anal. Chim. Acta.

[9] González-Ruiz V., Schvartz D., Sandström J., Pezzatti J., Jeanneret F., Tonoli D., Boccard J., Monnet-Tschudi F., Sanchez J.-C., Rudaz S. (2019). An Integrative Multi-Omics Workflow to Address Multifactorial Toxicology Experiments. Metabolites.

[10] Kalkhof S., Dautel F., Loguercio S., Baumann S., Trump S., Jungnickel H., Otto W., Rudzok S., Potratz S., Luch A. (2015). Pathway and Time-Resolved Benzo[a]Pyrene Toxicity on Hepa1c1c7 Cells at Toxic and Subtoxic Exposure. J. Proteome Res..

[11] Ritchie M.D., Holzinger E.R., Li R., Pendergrass S.A., Kim D. (2015). Methods of Integrating Data to Uncover Genotype–Phenotype Interactions. Nat. Rev. Genet..

[12] Lê Cao K.-A., Martin P.G., Robert-Granié C., Besse P. (2009). Sparse Canonical Methods for Biological Data Integration: Application to a Cross-Platform Study. BMC Bioinform..

[13] Wilms I., Croux C. (2016). Robust Sparse Canonical Correlation Analysis. BMC Syst. Biol..

[14] Hirai M.Y., Yano M., Goodenowe D.B., Kanaya S., Kimura T., Awazuhara M., Arita M., Fujiwara T., Saito K. (2004). Integration of Transcriptomics and Metabolomics for Understanding of Global Responses to Nutritional Stresses in Arabidopsis Thaliana. Proc. Natl. Acad. Sci. USA.

[15] Hirai M.Y., Klein M., Fujikawa Y., Yano M., Goodenowe D.B., Yamazaki Y., Kanaya S., Nakamura Y., Kitayama M., Suzuki H. (2005). Elucidation of Gene-to-Gene and Metabolite-to-Gene Networks in Arabidopsis by Integration of Metabolomics and Transcriptomics. J. Biol. Chem..

[16] Boccard J., Rutledge D.N. (2013). A Consensus Orthogonal Partial Least Squares Discriminant Analysis (OPLS-DA) Strategy for Multiblock Omics Data Fusion. Anal. Chim. Acta.

[17] El Bouhaddani S., Houwing-Duistermaat J., Salo P., Perola M., Jongbloed G., Uh H.-W. (2016). Evaluation of O2PLS in Omics Data Integration. BMC Bioinform..

[18] Brandolini-Bunlon M., Pétéra M., Gaudreau P., Comte B., Bougeard S., Pujos-Guillot E. (2019). Multi-Block PLS Discriminant Analysis for the Joint Analysis of Metabolomic and Epidemiological Data. Metabolomics.

[19] Bylesjö M., Eriksson D., Kusano M., Moritz T., Trygg J. (2007). Data Integration in Plant Biology: The O2PLS Method for Combined Modeling of Transcript and Metabolite Data. Plant J..

[20] Bylesjö M., Nilsson R., Srivastava V., Grönlund A., Johansson A.I., Jansson S., Karlsson J., Moritz T., Wingsle G., Trygg J. (2009). Integrated Analysis of Transcript, Protein and Metabolite Data To Study Lignin Biosynthesis in Hybrid Aspen. J. Proteome Res..

[21] Hassani S., Martens H., Qannari E.M., Hanafi M., Kohler A. (2012). Model Validation and Error Estimation in Multi-Block Partial Least Squares Regression. Chemom. Intell. Lab. Syst..

[22] Mehl F., Marti G., Merle P., Delort E., Baroux L., Sommer H., Wolfender J.-L., Rudaz S., Boccard J. (2015). Integrating Metabolomic Data from Multiple Analytical Platforms for a Comprehensive Characterisation of Lemon Essential Oils: Lemon Oil Characterisation by Multiblock Metabolomic Analysis. Flavour Fragr. J..

[23] Moyon T., Le Marec F., Qannari E.M., Vigneau E., Le Plain A., Courant F., Antignac J.-P., Parnet P., Alexandre-Gouabau M.-C. (2012). Statistical Strategies for Relating Metabolomics and Proteomics Data: A Real Case Study in Nutrition Research Area. Metabolomics.

[24] Alassane-Kpembi I., Gerez J.R., Cossalter A.-M., Neves M., Laffitte J., Naylies C., Lippi Y., Kolf-Clauw M., Bracarense A.P.L., Pinton P. (2017). Intestinal Toxicity of the Type B Trichothecene Mycotoxin Fusarenon-X: Whole Transcriptome Profiling Reveals New Signaling Pathways. Sci. Rep..

[25] Dellafiora L., Dall’Asta C. (2017). Forthcoming Challenges in Mycotoxins Toxicology Research for Safer Food—A Need for Multi-Omics Approach. Toxins.

[26] Pierron A., Mimoun S., Murate L.S., Loiseau N., Lippi Y., Bracarense A.-P.F.L., Liaubet L., Schatzmayr G., Berthiller F., Moll W.-D. (2016). Intestinal Toxicity of the Masked Mycotoxin Deoxynivalenol-3-β-d-Glucoside. Arch. Toxicol..

[27] Wu M., Xiao H., Ren W., Yin J., Hu J., Duan J., Liu G., Tan B., Xiong X., Oso A.O. (2014). An NMR-Based Metabolomic Approach to Investigate the Effects of Supplementation with Glutamic Acid in Piglets Challenged with Deoxynivalenol. PLoS ONE.

[28] Xiao H., Wu M.M., Shao F.Y., Tan B.E., Li T.J., Ren W.K., Yin J., Wang J., He Q.H., Yin Y.L. (2015). Metabolic Profiles in the Response to Supplementation with Composite Antimicrobial Peptides in Piglets Challenged with Deoxynivalenol1. J. Anim. Sci..

[29] Adeva-Andany M., López-Ojén M., Funcasta-Calderón R., Ameneiros-Rodríguez E., Donapetry-García C., Vila-Altesor M., Rodríguez-Seijas J. (2014). Comprehensive Review on Lactate Metabolism in Human Health. Mitochondrion.

[30] Huang N.-J., Lin Y.-C., Lin C.-Y., Pishesha N., Lewis C.A., Freinkman E., Farquharson C., Millán J.L., Lodish H. (2018). Enhanced Phosphocholine Metabolism Is Essential for Terminal Erythropoiesis. Blood.

[31] Newsholme E.A., Carrié A.L. (1994). Quantitative Aspects of Glucose and Glutamine Metabolism by Intestinal Cells. Gut.

[32] Adesso S., Autore G., Quaroni A., Popolo A., Severino L., Marzocco S. (2017). The Food Contaminants Nivalenol and Deoxynivalenol Induce Inflammation in Intestinal Epithelial Cells by Regulating Reactive Oxygen Species Release. Nutrients.

[33] Alassane-Kpembi I., Pinton P., Hupé J.-F., Neves M., Lippi Y., Combes S., Castex M., Oswald I. (2018). Saccharomyces Cerevisiae Boulardii Reduces the Deoxynivalenol-Induced Alteration of the Intestinal Transcriptome. Toxins.

[34] Boelaert J., Bendhaiba L., Olteanu M., Villa-Vialaneix N., Villmann T., Schleif F.-M., Kaden M., Lange M. (2014). SOMbrero: An R Package for Numeric and Non-numeric Self-Organizing Maps. Advances in Self-Organizing Maps and Learning Vector Quantization.

[35] Mariette J., Villa-Vialaneix N. (2017). Unsupervised Multiple Kernel Learning for Heterogeneous Data Integration. Bioinformatics.

[36] Payros D., Alassane-Kpembi I., Pierron A., Loiseau N., Pinton P., Oswald I.P. (2016). Toxicology of Deoxynivalenol and Its Acetylated and Modified Forms. Arch. Toxicol..

[37] Pizzorno J. (2014). Glutathione!. Integr. Med. Encinitas Calif.

[38] Zhou R., Tardivel A., Thorens B., Choi I., Tschopp J. (2010). Thioredoxin-Interacting Protein Links Oxidative Stress to Inflammasome Activation. Nat. Immunol..

[39] He F., Wu C., Li P., Li N., Zhang D., Zhu Q., Ren W., Peng Y. (2018). Functions and Signaling Pathways of Amino Acids in Intestinal Inflammation. BioMed Res. Int..

[40] Ravindran R., Loebbermann J., Nakaya H.I., Khan N., Ma H., Gama L., Machiah D.K., Lawson B., Hakimpour P., Wang Y.-C. (2016). The Amino Acid Sensor GCN2 Controls Gut Inflammation by Inhibiting Inflammasome Activation. Nature.

[41] Cano P.M., Seeboth J., Meurens F., Cognie J., Abrami R., Oswald I.P., Guzylack-Piriou L. (2013). Deoxynivalenol as a New Factor in the Persistence of Intestinal Inflammatory Diseases: An Emerging Hypothesis through Possible Modulation of Th17-Mediated Response. PLoS ONE.

[42] Edgar R. (2002). Gene Expression Omnibus: NCBI Gene Expression and Hybridization Array Data Repository. Nucleic Acids Res..

[43] R Core Team (2018). R: A Language and Environment for Statistical Computing. R Foundation for Statistical Computing.

[44] Gentleman R.C., Carey V.J., Bates D.M., Bolstad B., Dettling M., Dudoit S., Ellis B., Gautier L., Ge Y., Gentry J. (2004). Bioconductor: Open Software Development for Computational Biology and Bioinformatics. Genome Biol..

[45] Bolstad B.M., Irizarry R.A., Åstrand M., Speed T.P. (2003). A Comparison of Normalization Methods for High Density Oligonucleotide Array Data Based on Variance and Bias. Bioinformatics.

[46] Beckonert O., Keun H.C., Ebbels T.M.D., Bundy J., Holmes E., Lindon J.C., Nicholson J.K. (2007). Metabolic Profiling, Metabolomic and Metabonomic Procedures for NMR Spectroscopy of Urine, Plasma, Serum and Tissue Extracts. Nat. Protoc..

[47] Usal M., Veyrenc S., Darracq-Ghitalla-Ciock M., Regnault C., Sroda S., Fini J.-B., Canlet C., Tremblay-Franco M., Raveton M., Reynaud S. (2021). Transgenerational Metabolic Disorders and Reproduction Defects Induced by Benzo[a]Pyrene in Xenopus Tropicalis. Environ. Pollut..

[48] Cabaton N.J., Poupin N., Canlet C., Tremblay-Franco M., Audebert M., Cravedi J.-P., Riu A., Jourdan F., Zalko D. (2018). An Untargeted Metabolomics Approach to Investigate the Metabolic Modulations of HepG2 Cells Exposed to Low Doses of Bisphenol A and 17β-Estradiol. Front. Endocrinol..

[49] Cottret L., Frainay C., Chazalviel M., Cabanettes F., Gloaguen Y., Camenen E., Merlet B., Heux S., Portais J.-C., Poupin N. (2018). MetExplore: Collaborative Edition and Exploration of Metabolic Networks. Nucleic Acids Res..

[50] Kanehisa M., Furumichi M., Tanabe M., Sato Y., Morishima K. (2017). KEGG: New Perspectives on Genomes, Pathways, Diseases and Drugs. Nucleic Acids Res..

[51] Hotelling H. (1936). Relations between Two Sets of Variates. Biometrika.

[52] Kohonen T. (1982). Self-Organized Formation of Topologically Correct Feature Maps. Biol. Cybern..

[53] Olteanu M., Villa-Vialaneix N. (2015). Using SOMbrero for Clustering and Visualizing Graphs. J. Soc. Fr. Stat..

[54] Trygg J. (2002). O2-PLS for Qualitative and Quantitative Analysis in Multivariate Calibration. J. Chemom..

[55] Thévenot E.A., Roux A., Xu Y., Ezan E., Junot C. (2015). Analysis of the Human Adult Urinary Metabolome Variations with Age, Body Mass Index, and Gender by Implementing a Comprehensive Workflow for Univariate and OPLS Statistical Analyses. J. Proteome Res..

[56] Bylesjö M., Rantalainen M., Cloarec O., Nicholson J.K., Holmes E., Trygg J. (2006). OPLS Discriminant Analysis: Combining the Strengths of PLS-DA and SIMCA Classification. J. Chemom..

[57] El Bouhaddani S., Uh H.-W., Jongbloed G., Hayward C., Klarić L., Kiełbasa S.M., Houwing-Duistermaat J. (2018). Integrating Omics Datasets with the OmicsPLS Package. BMC Bioinform..

