# Supplementary material for: Statistical Integration of ‘Omics Data Increases Biological Knowledge Extracted from Metabolomics Data: Application to Intestinal Exposure to the Mycotoxin Deoxynivalenol
Source: Metabolites. 2021 Jun 21;11(6):407. doi: 10.3390/metabo11060407 (PMC8233929; doi:10.3390/metabo11060407)
Supplement: Supplementary file 1 [file metabolites-11-00407-s001.zip › Supplementary File S2.pdf]

Statistical integration of ‘omics data increases biological knowledge extracted from metabolomics data: application to intestinal exposure to the mycotoxin deoxynivalenol

Marie Tremblay Franco [1,2,*], Cécile Canlet [1,2], Philippe Pinton [1], Yannick Lippi, Roselyne Gautier [1,2], Claire Naylies [1], Manon Neves [1], Isabelle P. Oswald [1], Laurent Debrauwer [1,2] and Imourana Alassane-Kpembi [3]

[1] Toxalim (Research Center in food toxicology), Toulouse University, INRAE, ENVT, INP-Purpan, UPS, Toulouse, France; marie.tremblay-franco@inrae.fr; cecile.canlet@inrae.fr; philippe.pinton@inrae.fr; yannick.lippi @inrae.fr; roselyne.gautier @inrae.fr; claire.naylies@inrae.fr; manon.neves@inrae.fr; isabelle.oswald@inrae.fr; laurent.debrauwer@inrae.fr; iakpembi@gmail.com

[2] Metatoul-AXIOM platform, MetaboHUB, Toxalim, INRAE, Toulouse, France

[3] Department of Veterinary Biomedicine, Faculty of Veterinary Medicine, Université de Montréal, Saint-Hyacinthe, Québec, Canada

* Correspondence: marie.tremblay-franco@inrae.fr

Simulation study.

To assess the performance of the sparse CCA and SOM methods, we conducted a simulation study. We generated two datasets, X and Y, corresponding, respectively, to transcriptomic and metabolomic data. To mimic biological data, we used several numbers of features, from 1000 and 100 to 12,000 and 775 for, respectively, transcriptomic and metabolomic matrices. A subset of variables in X was associated with a subset of variables in Y, and the remaining variables were simulated as noise. Simulated datasets consisted of ten individuals. This simulation study enabled us to assess if the sparse CCA and SOM methods can differentiate correlated variables from noisy variables.

With the aim to generate an artificially simulated dataset, we used a real dataset to keep the baseline sources of variability. We used microarray transcriptomic data and NMR metabolomic data generated from ten individuals of an independent study that had previously been published (Montagner et al., 2016). Then, we applied several steps to both datasets to define noisy features and add artificial effects to selected features.

1. Subtraction of known sources of experimental variability using the RemoveBatchEffect function from the limma R package (Ritchie et al., 2015).
2. Computation of inter and intra correlation (Pearson method) matrices between transcriptomic and metabolomic matrices. The obtained correlation coefficients help us define “correlated” (cor>=0.9) vs. “uncorrelated” (cor<0.9) features.
3. Random simulation of datasets following the above steps:
   3.1. Random draw of 0.1*p “correlated” transcriptomic and 0.1*q “correlated” metabolomic features.
   3.2. Random draw of 0.9*p “uncorrelated” transcriptomic and “uncorrelated” 0.9*q metabolomic features.
   3.3. Generation of noisy features based on the multivariate normal distribution N(0, Σ).
   3.4. Addition of four artificial experimental effects (+3, -3, +1.3 and -1.3) to mimic biological condition to pFBio transcriptomic and qFBio metabolomic (respectively) “uncorrelated” and “correlated” features for half of the individuals (randomly sampled).

The simulation scheme (steps 3.1 to 3.4) was repeated 100 times for each of 4 dataset dimensions (big, large, medium, and small).

Table S1: Simulation design.

	Transcriptomic dataset		Metabolomic dataset	
Simulated dataset dimensions	Number of variables (p)	Number of variables with biological effect (pFBio)	Number of variables (q)	Number of variables with biological effect (qFBio)
big	12000	100	788	10
large	10000	85	700	10
medium	5000	40	500	5
small	1000	10	100	2

The sparse CCA and SOM methods were evaluated using sensibility and specificity measures. Sensitivity (Se) corresponds to the proportion among correlated features (within and between ‘omic datasets) that were declared correlated. Specificity (Sp) corresponds to the proportion among non-correlated features that were declared non-correlated (and are rightly not correlated). The sensitivity is a measure of how well the method is able to correctly identify correlated features, while the specificity measures how well the method can identify uncorrelated features. With binary decisions (correlated/uncorrelated), results can be displayed in a 2 x 2 table that cross classifies true status (e.g.,

features are correlated, yes/no) with predicted status (e.g., features are predicted to be correlated, yes/no), summarizing the performance of the method. The sensitivity and specificity are computed from Table S2.

Table 2: Confusion matrix used to compute sensitivity and specificity.

Method \ Truth	Correlated	Uncorrelated
Correlated	True positive (TP)	False positive (FP)
Uncorrelated	False negative (FN)	True negative (TN)

Se = TP/(TP+FN) et Sp = TN/(FP+TN).

For the SOM method, a pair of features was considered as correlated when both features were classified into the same unit of the map.

The R^2 value and the Mean Squared Error of Prediction (MSEP) were used to assess the performance of the O2PLS models. R^2 corresponds to the explained variance. MSEP (Mevik and Cederkvist, 2004) measures how well a model predicts. It is based on the squared difference in the predicted and observed values of the biological factor. The lower the MSEP is, the better predicts the model. MSEP was computed using leave-one-out cross-validation.
