# Supplementary material for: Statistical Integration of ‘Omics Data Increases Biological Knowledge Extracted from Metabolomics Data: Application to Intestinal Exposure to the Mycotoxin Deoxynivalenol
Source: Metabolites. 2021 Jun 21;11(6):407. doi: 10.3390/metabo11060407 (PMC8233929; doi:10.3390/metabo11060407)
Supplement: Supplementary file 1 [file metabolites-11-00407-s001.zip › metabolites-1224058 sup proof.pdf]

Statistical Integration of 'Omics Data Increases Biological Knowledge Extracted from Metabolomics Data: Application to Intestinal Exposure to the Mycotoxin Deoxynivalenol

Marie Tremblay Franco [1,2,*], Cécile Canlet [1,2], Philippe Pinton [1], Yannick Lippi [1], Roselyne Gautier [1,2], Claire Naylies [1], Manon Neves [1], Isabelle P. Oswald [1], Laurent Debrauwer [1,2] and Imourana Alassane-Kpembi [3]

[1] Toxalim (Research Center in Food Toxicology), Toulouse University, INRAE, ENVT, INP-Purpan, UPS,
F-31027 Toulouse, France; cecile.canlet@inrae.fr (C.C.); philippe.pinton@inrae.fr (P.P.); yannick.lippi@inrae.fr (Y.L.); roselyne.gautier@inrae.fr (R.G.); claire.naylies@inrae.fr (C.N.); manon.neves@inrae.fr (M.N.); isabelle.oswald@inrae.fr (I.P.O.); laurent.debrauwer@inrae.fr (L.D.)

[2] Metatoul-AXIOM Platform, MetaboHUB, Toxalim, INRAE, F-31027 Toulouse, France

[3] Department of Veterinary Biomedicine, Faculty of Veterinary Medicine, Université de Montréal, Saint-Hyacinthe, QC J2S 2M2 , Canada; imourana.alassane-kpembi@umontreal.ca

* Correspondence: marie.tremblay-franco@inrae.fr

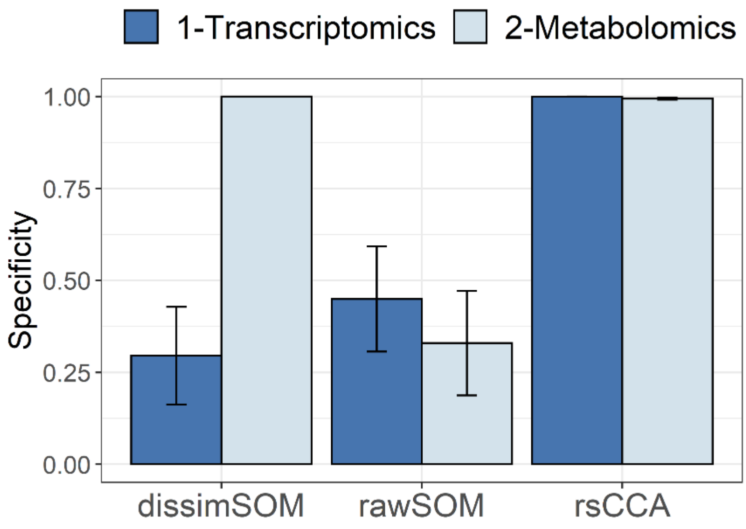

Figure S1. Average specificity of SOM (dissimilarity kernel-based and raw) and Robust Sparse CCA methods calculated for transcriptomic (middle blue bars) and metabolomic (light blue bars) blocks. One hundred Monte Carlo simulations.

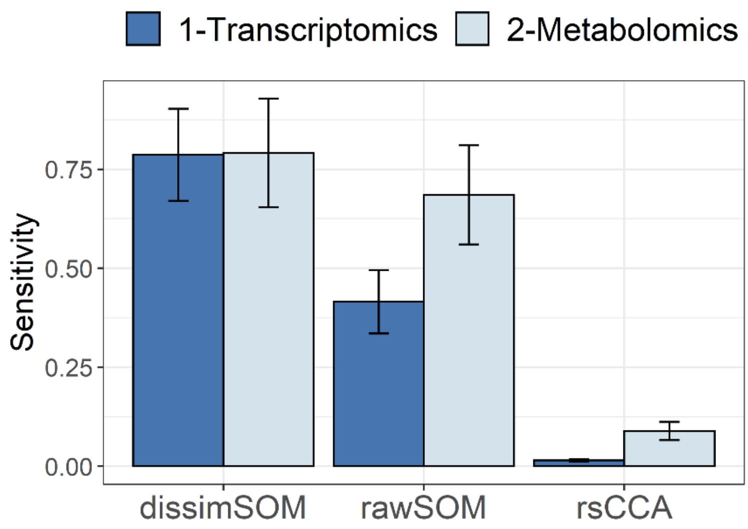

Figure S2. Average sensitivity of SOM (dissimilarity kernel-based and raw) and Robust Sparse CCA methods calculated for transcriptomic (middle blue bars) and metabolomic (light blue bars) blocks. One hundred Monte Carlo simulations.

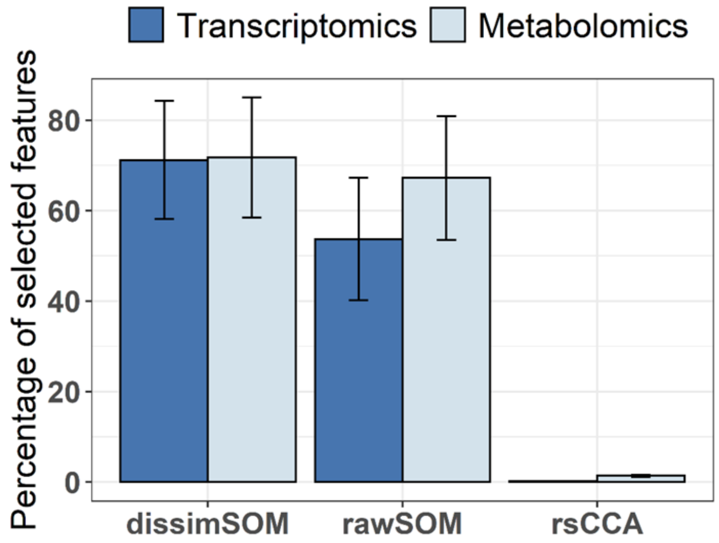

Figure S3. Average percentage of features selected by SOM (dissimilarity kernel-based and raw) and Robust Sparse CCA methods for transcriptomic (middle blue bars) and metabolomic (light blue bars) blocks. One hundred Monte Carlo simulations.
